# First Honorary Medal of the Signal Transduction Society (STS) and 'CELL COMMUNICATION AND SIGNALING' awarded to Professor Anthony J. (Tony) Pawson

**DOI:** 10.1186/1478-811X-9-3

**Published:** 2011-01-19

**Authors:** Stephan M Feller

**Affiliations:** 1 Cell Signalling Group, Weatherall Institute of Molecular Medicine, University of Oxford, Oxford OX3 9DS, UK

## Editorial

The 14^th ^annual gathering of the Signal Transduction Society (STS; http://www.sigtrans.de) brought together scientists from eleven different countries in Weimar, Germany. The conference focus topic for 2010 was *'Signal Transduction, Drug Discovery and Design'*.

Accordingly, the molecularly targeted interference with disease-driving signal transduction pathways was a major theme of many presentations. From these it became apparent that, in addition to the by now almost 'classical' blockers of kinases, inhibitors of protein - protein interactions are beginning to make their way towards the center stage.

It was therefore very timely that the newly introduced Honorary Medal of STS and CCS was presented to Prof. Tony Pawson, one of the founding fathers of protein - protein interaction research in cell signaling 'for the discovery of protein interaction domains and elucidating their essential roles in the transmission of cellular signals'.

Tony and his team discovered the Src Homology 2 (SH2) domains in the mid 1980s as conserved and functionally relevant regions in cytoplasmic tyrosine kinases [1,2]. From there, it took almost another five years until the ability of SH2 domains to bind to certain tyrosine phosphorylated proteins was reported by three groups, initially at the 6^th ^Oncogene Meeting (26-30 June 1990; Frederick, MD, USA) [3-5].

From then on this research field expanded massively and Tony Pawson has been repeatedly leading the way, conceptually and experimentally. At present over 200 posttranslational protein modifications and over 100 'reader' domains for these modifications have been identified in the human proteome.

One of several conference highlights in this area was the presentation of multiple novel crystal structures and an anti-oncogenic inhibitor of BET subfamily bromodomains by Panagis Filippakopoulos et al. from the Oxford branch of the Structural Genomics Consortium [6]. This first example of a specific inhibitor for a chromatin modification-reading protein interaction domain with *in vivo *activity is expected to add further momentum to the current shift of interests in the pharmaceutical sector towards the realm of epigenetic processes.

Another important message emerged, for example, from the presentation of Toby Gibson (EMBL, Heidelberg): it is time for signal transduction researchers to say 'Goodbye' to simple cascade models, and also to mathematical equations based on solution phase chemistry, which are often useless or misleading if one wants to quantitatively describe the actions of signal transduction proteins that act as parts of large multi-protein complexes [7]. Moreover, a bit like the internet, the signal transduction systems of cells appear to have no true master controllers, presumably giving them greater robustness against various forms of assault and also providing adaptability to new environmental challenges that may not be as feasible or effective with more hierarchical networks.

Clearly, signal transduction research has come a long way since its early beginnings, but new challenges are readily awaiting attention, especially with respect to understanding higher levels of signal processing, signal fine-tuning, and also the elucidation of spatio-temporal details and mechanisms of action of physiological signaling events, for example by high resolution imaging in real-time.
